# Human Rabies Deaths — Minnesota and California, 2024

**DOI:** 10.15585/mmwr.mm7502a4

**Published:** 2026-01-15

**Authors:** Malia Ireland, Curtis L. Fritz, Stacy Holzbauer, Carrie Klumb, Maria Bye, Leah Bauck, Amanda Bakken, Michelle Dethloff, Rebecca Campagna, Mojgan Deldari, Susan Hepp, Ruth Lopez, Sharon Messenger, Christopher Preas, Aimee Rendon, Stephanie Koch-Kumar, Maria Rangel, Kanwaldeep Bains, Jeffrey Bulawit, Samer Al Saghbini, Trinidad Solis, Joe Prado, John Zweifler, Leticia Berber, Rais Vohra, Ignacio A. Santana, Salvador Sandoval, Parmjit Sahota, Mark Hendrickson, Megan Black, Gloria Chavez, Andrew Schwab, Pamela A. Yager, Michael Niezgoda, Vaughn V. Wicker, Crystal M. Gigante, Lillian A. Orciari, Jesse D. Blanton, Sarah C. Bonaparte, Rebecca Earnest, Ryan M. Wallace

**Affiliations:** ^1^Minnesota Department of Health; ^2^California Department of Public Health, Richmond and Sacramento, California; ^3^North Dakota Department of Health; ^4^Fresno County Department of Public Health, Fresno, California; ^5^Merced County Department of Health, Merced, California; ^6^Division of High-Consequence Pathogens and Pathology, National Center for Emerging and Zoonotic Diseases, CDC; ^7^Oak Ridge Associated Universities, Oak Ridge, Tennessee; ^8^Epidemic Intelligence Service, CDC.

**Summary**

**What is already known about this topic?**

Rabies virus is maintained in wild mammals in the continental United States and is typically transmitted through bites from infected animals. Rabies is nearly universally fatal without administration of timely postexposure prophylaxis (PEP).

**What is added by this report?**

CDC confirmed two deaths of U.S. residents from rabies virus infection after bat encounters in 2024. Both patients recognized their bat interaction; however, they might not have been aware of the potential rabies risk, and neither sought health care consultation, bat testing, or PEP.

**What are the implications for public health practice?**

Increased awareness of the potential rabies risk after any bat encounter, even without a visible bite wound, might help prevent deaths.

## Abstract

Rabies is an enzootic viral disease in the continental United States and is typically transmitted through the bite of an infected mammal. Infection is almost always fatal if rabies postexposure prophylaxis (PEP) is not received before the onset of symptoms. Bats are the leading source of U.S. human rabies cases. In 2024, CDC identified two U.S. human rabies deaths in September (Minnesota) and November (California) in persons who had a recognized bat encounter but might not have been aware of the potential rabies risk. Neither patient reported the bat encounter to public health officials nor sought medical attention, including PEP, before symptom onset. Health officials conducted risk assessments among 384 persons in Minnesota, North Dakota, and California who had possible contact with either the bats that were presumed to have rabies or the patients while they were infectious; 45 (12%) of these persons were recommended to receive PEP. Bat bites often result in trivialized or inapparent wounds. Anyone with a possible bat exposure, even in the absence of a recognized bite, should immediately report the encounter to a health care provider or to public health officials for risk assessment, consideration of options for bat testing, and PEP administration, if indicated. Increased awareness of the potential risk for rabies after any bat interaction, even without a visible bite wound, might help prevent deaths.

## Introduction

Although rabies is enzootic in the continental United States and is typically transmitted through bites from infected mammals, human rabies deaths in this country are rare (*1*). Each year, among an estimated 1.4 million persons in the United States who seek medical care after animal contact, 100,000 (7%) receiverabiespostexposureprophylaxis(PEP). Rabies is nearly universally fatal if PEP is not administered before symptom onset. PEP is not indicated when the animal test results are negative or when public health officials determine that the contact does not pose a rabies risk (*2*). Bat exposures are the leading source of U.S. human rabies cases: among the 42 U.S.-acquired human rabies cases reported during 2000–2024, bat contact was the cause in 35 (83%) (*1*).

In September and November 2024, two human rabies deaths associated with bat contact in Minnesota and California, respectively, were reported to CDC. Although the Advisory Committee on Immunization Practices (ACIP) recommends that anyone with possible bat contact receive a rabies exposure risk assessment to ascertain the need for PEP, both of these deaths occurred in persons who, although aware of their bat encounter, did not consult with medical professionals or public health officials or receive PEP before symptom onset. CDC (and the California state laboratory for the California case) provided human rabies diagnostic testing. After confirming the rabies diagnosis, state and local health departments led the resulting animal exposure and patient contact investigations, with technical assistance provided by CDC. This report describes the characteristics and outcomes of these two fatal cases. This activity was reviewed by CDC, deemed not research, and was conducted consistent with applicable federal law and CDC policy.^†^

## Investigation and Outcomes

### Minnesota Case, July–September 2024

**Bat encounter.** In July 2024, a Minnesota woman who lived alone reported to family members that a bat or bird had been trapped in her house for several days. After discovering a bat in the sink, she reportedly killed it with a hammer and disposed of it. A bite was not mentioned; however, the method reportedly used to kill the bat could have produced splatter resulting in inoculation of infectious nervous tissue onto broken skin or mucous membranes. In addition, family members reported that the patient wore a hearing aid, was a deep sleeper who used a continuous positive airway pressure machine, and routinely consumed alcohol, factors that might have reduced her awareness of having had direct bat contact. Public health officials were not notified about the possible exposure, and the bat was not tested for rabies.

**Clinical course and rabies diagnosis.** In August, approximately 3 weeks after the bat encounter, the patient developed shoulder pain and weakness. During the next 9 days, she sought care several times for malaise, weakness, and continued shoulder pain; her medical record contains no documentation that she reported the bat encounter at any of those health care assessments. Ten days after initial symptom onset, she returned to a Minnesota hospital emergency department with tremors, progressive weakness, confusion, anxiety, and muscular rigidity. The patient was admitted to the hospital for supportive care and diagnostic testing, which included a lumbar puncture, a head and cervical spine computed tomography scan, and a multiplex polymerase chain reaction meningitis/encephalitis panel.^§^ On the first hospital day, the patient experienced an acute mental status change and was found to be minimally responsive, resulting in emergency endotracheal intubation and transfer to the intensive care unit. Family members reported the patient’s bat encounter at that time, and rabies was considered, but public health officials were not consulted regarding diagnostic testing because test results for more common diagnoses were pending. On the second hospital day, the patient was transferred to a tertiary care hospital in North Dakota, where providers noted signs of encephalitis. Twelve days after admission, the encephalopathy had not resolved, and the patient’s family elected to provide only palliative care. However, because extensive testing while the patient was hospitalized had still not identified a pathogen, after a consultation with state public health officials and CDC, a limited number of remaining antemortem samples (serum, cerebrospinal fluid, and plasma) were sent to CDC for rabies diagnostic testing. Later that day, the patient died, and her family declined both an autopsy and postmortem sampling for additional rabies testing. Rabies virus antibodies were detected in a plasma sample, confirming a diagnosis of rabies.

### California Case, October–November 2024

**Bat encounter**. In October 2024, a woman living in California told family members that she had recently found a bat indoors at her worksite. Although the bat initially appeared to be dead, when she handled it with her bare hands, she felt movement and a possible bite. She discarded the bat, and in the absence of any apparent wound, did not consult a medical provider or public health officials, and the bat was not tested for rabies.

**Clinical course and rabies diagnosis.** Approximately 1 month after the bat encounter, the patient developed paresthesia and muscle spasms in her arm. Three days later, she was hospitalized with seizures. On admission, the patient’s bat exposure was disclosed (whether this information was reported by the patient or family members is not known), prompting medical providers to contact public health officials regarding rabies testing. On the same day, the patient’s seizure activity worsened and was followed by mental status changes, leading to endotracheal intubation and transfer to the intensive care unit. During the following 3 days, her condition deteriorated, ultimately progressing to liver and kidney failure. The patient died 4 days after admission, and the California state laboratory and CDC confirmed a diagnosis of rabies through detection of rabies virus antigen in an antemortem nuchal skin biopsy and viral RNA in antemortem nuchal skin biopsy and saliva. Viral sequencing confirmed a bat rabies virus variant.

## Public Health Response

### Identification of Exposed Contacts

The detection of each human rabies case prompted an investigation to 1) determine the exposure circumstances, 2) identify other persons possibly exposed to the same animal that was presumed to be rabid, and 3) identify persons exposed to the patients during their infectious period.

A rabies exposure is defined as direct contact between broken skin or mucous membranes and the tears, saliva, or nervous tissues of an infected animal or person. Health departments administered a risk assessment questionnaire to any person who had possible contact with either the bats that were presumed to have rabies or with the patients while they were possibly infectious; based on limited available data on duration of viral shedding, the infectious period was conservatively estimated to be 14 days before symptom onset until death and decontamination (*3*). These assessments included 155 persons in Minnesota, 185 in North Dakota, and 44 in California. All of the potential exposures were to the patients; no bat exposures were identified.

### PEP Recommendations for Identified Exposed Contacts

Among 155 assessed persons in Minnesota, five of 35 (14%) community contacts and nine of 120 (8%) health care worker contacts were recommended to receive PEP (Table). In North Dakota, all 185 assessed persons were health care workers, 23 (12%) of whom were recommended to receive PEP. In California, among assessed persons, two of six community contacts and six of 38 (16%) health care workers were recommendedtoreceivePEP. Across both patient investigations, PEP was recommended for a total of 45 (12%) of 384 exposed persons, including seven (17%) of 41 community contacts and 38 (11%) of 343 health care worker contacts; information regarding receipt and completion of PEP is not available.

**TABLE:** Health department assessment of contacts of two patients with rabies* and number of contacts^†^ recommended to receive rabies postexposure prophylaxis,^§^^,¶^ by contact type — Minnesota, North Dakota, and California, 2024

State	Contacts, no. (%)					
	Community		Health care workers		Total	
	Assessed	PEP recommended	Assessed	PEP recommended	Assessed	PEP recommended
Minnesota	35	5 (14)	120	9 (8)	**155**	**14 (9)**
North Dakota	0	NA	185	23 (12)	**185**	**23 (12)**
California	6	2 (33)	38	6 (16)	**44**	**8 (18)**
**Total**	**41**	**7 (17)**	**343**	**38 (11)**	**384**	**45 (12)**

**Abbreviations:** NA = not applicable; PEP = postexposure prophylaxis.

* Both human rabies cases resulted from contact with bats.

**^†^** All potential exposures were to the patients; no contact was exposed to the bats that were presumed to have rabies.

**^§^** PatientCareforPreventingRabies|CDC

^¶^ Information regarding receipt or completion of PEP is not available.

### Public Health Recommendations Regarding Bat Exposures

Press releases about the rabies cases were issued by health departments in Minnesota and California. The press releases included rabies prevention messaging focused on the risks from bats and the importance of consulting health care providers or public health officials about bat contact or encounters, even in the absence of a recognized bite.

## Discussion

Bats are the leading source of human rabies cases in the United States, largely because bat bites often result in trivialized or inapparent wounds (*4*). North American bat species are relatively small, and their bite wounds can be difficult to detect. The California case described in this report highlights the importance of reporting bat encounters, even when a bite or scratch is inapparent, and reinforces current ACIP guidance regarding bat handling. Bats should never be handled with bare hands; CDCadvises wearing leather or bite-proof gloves when handling any bat. Furthermore, sick bats might appear dead and are more likely to be infected with pathogens, including rabies virus, than are apparently healthy bats. Bats are a critical part of the ecosystem, and healthy bats typically avoid human contact. For this reason, ACIP recommends a rabies risk assessment by a health care provider or public health professional for any direct bat contact and that PEP be administered in situations when a bat bite or scratch cannot be ruled out and the animal is unavailable for rabies testing (*5*).

Although most healthy persons would likely detect direct physical contact with a bat, certain conditions have been noted to increase the risk for unrecognized bat exposures, leading to rabies virus transmission. These conditions include reduced mental capacity or age-related factors that would affect awareness or ability to communicate an exposure; use of drugs, alcohol, or medications that could reduce perception of bat contact; and a tendency to sleep through noises or disturbances that typically awaken others, including contact with a bat (*5*). Therefore, ACIP recommends that persons who have slept in a room where a bat is present and are at increased risk for unrecognized exposure should receive PEP (*5*). Although the Minnesota patient described in this report was aware of a bat in her home, multiple characteristics that could have reduced her perception of direct bat contact were noted, including that she wore a hearing aid, was reported to be a deep sleeper who used a continuous positive airway pressure machine, and routinely consumed alcohol, and thereby would have met the criteria for PEP as described by ACIP.

### Implications for Public Health Practice

At least 44 bat species are found in the continental United States (*6*), and rabies virus has been detected across nearly all species that have undergone testing (*1*). Among bats submitted for rabies testing in the United States, approximately 5% have been found to be infected with rabies virus (*1*). Given the prevalence of rabies virus among domestic bat species, the nearly universally fatal nature of rabies disease, and the risk for trivialized or unperceived exposures, persons should be vigilant for bats in occupied buildings and immediately report encounters to health care providers or public health officials for risk assessment, animal testing options, and PEP administration, if indicated. Increased public awareness of the potential rabies risk after any bat encounter, including those that do not result in visible wounds, might prevent deaths.

## Footnotes

* These authors contributed equally to this report.

† 45 C.F.R. part 46, 21 C.F.R. part 56; 42 U.S.C. Sect. 241(d); 5 U.S.C. Sect. 552a; 44 U.S.C. Sect. 3501 et seq.

§ The multiplex polymerase chain reaction meningitis/encephalitis panel tests for 14 pathogens in a cerebrospinal fluid sample to rule out more common causes of meningitis or encephalitis; rabies virus is not included in the panel.
